# Secretomes of human pluripotent stem cell-derived smooth muscle cell progenitors upregulate extracellular matrix metabolism in the lower urinary tract and vagina

**DOI:** 10.1186/s13287-021-02292-y

**Published:** 2021-04-06

**Authors:** Guobing Zhuang, Yan Wen, Mason Briggs, Qingchun Shao, Darlene Tran, Hongbo Wang, Bertha Chen

**Affiliations:** 1 Department of Obstetrics/Gynecology, Stanford University School of Medicine, 300 Pasteur Drive HH-333, Stanford, CA 94305 USA; 2 Department of Obstetrics/Gynecology, Union Hospital, Tongji Medical College, Huazhong University of Science and Technology, Wuhan, People’s Republic of China

**Keywords:** Secretome, Smooth muscle cell progenitors, Pluripotent stem cells, iPSC, Stress urinary incontinence

## Abstract

**Background:**

Adult mesenchymal stem cells (MSCs) have been studied extensively for regenerative medicine; however, they have limited proliferation in vitro, and the long culture time induces cell senescence. MSCs also contribute to tissue repair through their paracrine function. In this study, we sought to examine the paracrine effects of human smooth muscle cell progenitors (pSMC) on the urethra and adjacent vagina of stress urinary incontinence rodents. We use human pluripotent stem cell (PSC) lines to derive pSMCs to overcome the issue of decreased proliferation in tissue culture and to obtain a homogenous cell population.

**Method:**

Three human PSC lines were differentiated into pSMCs. The conditioned medium (CM) from pSMC culture, which contain pSMC secretomes, was harvested. To examine the effect of the CM on the extracellular matrix of the lower urinary tract, human bladder smooth muscle cells (bSMCs) and vaginal fibroblasts were treated with pSMC-CM in vitro. Stress urinary incontinence (SUI) was induced in rats by surgical injury of the urethra and adjacent vagina. SUI rats were treated with pSMC-CM and monitored for 5 weeks. Urethral pressure testing was performed prior to euthanasia, and tissues were harvested for PCR, Western blot, and histological staining. Kruskal-Wallis one-way ANOVA test and Student *t* test were used for statistical comparisons.

**Results:**

pSMC-CM upregulated MMP-2, TIMP-2, collagen, and elastin gene expression, and MMP-9 activity in the human bladder and vaginal cells consistent with elastin metabolism modulation. pSMC-CM treatment in the SUI rat improved urethral pressure (increase in leak point pressure compared to intact controls, *p* < 0.05) and increased collagen and elastin expression in the urethra and the adjacent vagina.

**Conclusion:**

Conditioned media from smooth muscle cell progenitors derived from human pluripotent stem cells improved urethral leak point pressure and collagen and elastin content in the SUI rat. These findings suggest a novel therapeutic potential for PSC-based treatments for SUI and pelvic floor disorders where tissues are affected by collagen, elastin, and smooth muscle loss.

**Supplementary Information:**

The online version contains supplementary material available at 10.1186/s13287-021-02292-y.

## Background

Stress urinary incontinence (SUI) is one of the most prevalent pelvic floor disorders affecting many adults worldwide [1]. SUI is defined by an involuntary loss of urine during physical effort and its etiology is thought to be multifactorial [2]. Pregnancy, vaginal delivery, trauma, pelvic surgery, and aging are among the list of factors associated with the development of SUI [3–5]. Histologically, what is observed is a loss of muscular structure and cells in the urethral sphincter with a shift to collagenous tissue, as well as vascular and nerval changes in tissue of the lower urinary tract [6]. Current treatment options include pelvic floor physical therapy [7], bulking agent injections into the urethral tissue, or surgery with placement of synthetic mesh sling around the mid-urethra to provide support [8–10]. Surgical treatment is efficacious but the adverse events, including general risks of surgery, synthetic mesh complications, nerve injury, bladder perforation, postoperative voiding dysfunction [11, 12], and long-term recurrence rates, have led to a quest for the development of new approaches.

Data from animal models using animal autologous mesenchymal stem cells (MSC) transplanted into the urethra suggest that these cells may assist tissue repair through secretion of trophic factors, rather than by significant cell regeneration resulting from in vivo differentiation of the MSC into terminally differentiated muscle cells [13]. Furthermore, several studies on stem cell-derived secretory factors suggest that the secreted factors alone, without the presence of the stem cells, may be sufficient to enhance tissue repair after injury [14, 15]. The secreted factors are referred to as secretome, microvesicles, or exosome and can be found in the medium where the stem cells are cultured, also called conditioned medium (CM) [16].

Human-induced pluripotent stem cells (hiPSCs) have also been explored as a stem cell source for treatment of SUI. A unique advantage of these cells over the adult MSCs is that they can be expanded in large quantities and differentiated in vitro into autologous organ-specific cells using defined and reproducible protocols [17, 18]. Expansion of adult MSCs can be limited by availability, decreased proliferation, and cell senescence. Our previous study indicated that periurethral transplantation of hiPSC-derived smooth muscle cell progenitors (pSMCs) promote functional restoration of the injured rat urethra through pSMC engraftment into the tissues [19]. We also observed significant changes in the native rodent tissue extracellular matrix (ECM) in the pSMC transplanted urethras with a shift to increased elastin deposition rather than the stiff, collagenous tissue typical of chronic injury.

Given mounting data on the anti-fibrotic effects of MSC conditioned media [20], and our observations of in vivo changes in rodent ECM with human pSMC transplantation, we hypothesize that the pSMC-conditioned media (pSMC-CM) derived from human pluripotent stem cells will also modulate ECM in the urethra and adjacent vagina to restore urethral function in the SUI rodent model. In the present study, we sought to characterize the effect of pSMC-CM on ECM metabolism in human lower urinary tract and vaginal cells in vitro and examine the in vivo regenerative effects of pSMC-CM on the urethra and vagina of the SUI rodent model. These proof-of-concept studies on the effect of progenitor cell secretomes will help expand the therapeutic potential of pluripotent stem cell-based therapies for SUI and for other pelvic floor disorders associated with deficient tissues.

## Methods

### Cell culture

This study was approved by the Institutional Review Board of the Stanford University School of Medicine and the Stanford University Stem Cell Research Oversight Committee. Three human pluripotent stem cell (PSC) lines were used in this study for differentiation into pSMCs: (1) Huf5-iPSC line was reprogrammed from female (premenopause) dermal fibroblasts via viral transduction of the transcription factors Oct3/4, Sox2, Klf4, and c-Myc [21]; (2) BIR-iPSC line was reprogrammed from female (premenopause) dermal fibroblasts with modified miRNA reprogramming method [22]. mRNA encoding Oct4, Klf4, Sox2, c-Myc, and Lin28 were co-transfected with a mixture of several miRNAs into the fibroblasts to induce iPSCs; and (3) H9 human embryonic stem cell line was a gift from Dr. Joseph Wu, Stanford University. All PSC lines were cultured on Matrigel-coated dishes (BD Biosciences, San Diego, CA) in mTeSR medium (StemCell Technologies, Vancouver, BC).

Human bladder smooth muscle cells (bSMCs) were isolated from three female donors (30–50 years old which were labeled as B1, B2, and B3, respectively). IRB exemption and external approval from Donor Network West’s (federally designated organ procurement organization) Internal Research Council and Medical Advisory Board Research Subcommittee was obtained for collection of primary bladder tissue from the three female transplant donors. Patients with history of bladder pathology were excluded from study. The muscular layer of fresh bladders was cut into 2mm × 2mm pieces and cultured in 80% Dulbecco’s modified Eagle’s medium (DMEM; Invitrogen, Carlsbad, CA) and 20% fetal bovine serum (Invitrogen) at 37 °C in an atmosphere of 95% air and 5% CO_2_. Tissue fragments were removed on day 14 and culture medium was changed to 90% DMEM and 10% FBS. Cells were passed to next passage with 0.05% Trypsin with EDTA (Thermo Scientific, Fremont, CA) when they were 80–90% confluent. SMC markers including αSMA and SM22 were detected by immunofluorescent staining to confirm cell types (data not shown).

The informed consents were obtained from all donors for isolating human vaginal wall fibroblasts. Human vaginal fibroblasts were isolated from three female donors which were labeled as F1, F2, and F3 (age 60–70 years old), respectively. Fibroblast cell explants were cut into approximately 1 mm × 1mm fragments and cultured in 80% Dulbecco’s modified Eagle’s medium (DMEM; Invitrogen) and 20% fetal bovine serum (Invitrogen) at 37 °C in an atmosphere of 95% air and 5% CO_2_ [23]. Tissue fragments were removed on day 14 and culture medium was changed into 90% DMEM and 10% FBS. Cells were passed to next passage with 0.05% Trypsin with EDTA (Thermo Scientific, Fremont, CA) when they were 80–90% confluent.

### Directed differentiation of human pluripotent stem cells to pSMCs and conditioned medium collection

Human pSMCs were differentiated from the three pluripotent stem cell lines using a modified, feeder cell-free, vascular progenitor protocol [22, 24, 25]. Briefly, the stem cells were seeded at a density of 5000–20,000 cells per cm^2^ of a 10-cm dish precoated with Matrigel in mTeSR supplemented with Thiazovivin (2 μM; Cayman Chemical Company, Ann Arbor, MI). After 24–72 h in cultured in chemical defined medium (RPMI 1640 with 1 mM glutamax, 1% nonessential amino acids, 0.1 mM β-mercaptoethanol, 1% penicillin, and streptomycin [Life Science Technology, Inc.], 1% ITS [Corning, Tewksbury, MA]) supplemented with Activin A (50 ng/mL), BMP4 (50 ng/mL; PeproTech, Rocky Hill, NY), and 2.5 μM/mL GSK 3 inhibitor, CHIR99021 on day 0, 2.0 μM/mL CHIR99021 on day 1 (Cayman Chemical Company), followed by basic fibroblast growth factor (50 ng/mL) and vascular endothelial growth factor (40 ng/mL; PeproTech) from day 2 to 12–14 days.

The differentiated cells were dissociated with Accutase (Innovative Cell Technologies, San Diego, CA) and prepared for magnetic-activated cell sorting (MACS) and fluorescence-activated cell sorting (FACS) of CD31^+^/CD34^+^ vascular progenitor cells (VPCs) [26]. Briefly, the cells were first immunolabeled with CD34 microbeads for MACS (Miltenyi Biotec, Auburn, CA), and magnetic labeling was performed according to the manufacturer’s instruction. The magnetically labeled CD34^+^ VPCs were obtained by positive selection. The CD34^+^ VPCs sorted by MACS were immediately blocked with mouse IgG (R&d Systems, Inc., Minneapolis, MN) for 10 min and stained with FITC mouse anti-human CD31 and PerCP-Cy5.5 mouse anti-human CD34 antibodies (BD biosciences) for 30 min at 4 °C. The CD34+/CD31+ cells were sorted on a FACS Aria II (BD Biosciences). After sorting, the cells, which were now labeled as passage 0 (P0), were expanded on mouse collagen IV-precoated plates (BD Biosciences) in smooth muscle cell growth medium (SMGS; Invitrogen) supplemented with recombinant human platelet-derived growth factor-BB (10 ng/mL, PDGF-BB; PeproTech) to yield pSMCs. The pSMCs were passed with 0.05% Trypsin with EDTA (Thermo Scientific, Fremont, CA) when they reached 80–90% confluency until they were harvested at passage 3 (P3). Conditioned medium was collected and frozen at − 80 °C when medium was changed every other day from P0 to P3. The conditioned medium for each cell line was pooled at P0 and at P3, and a small amount of the pooled conditioned medium from each passage was frozen at − 80 °C. The conditioned medium was collected and pooled at P4 for each cell line and concentrated 50 times by ultrafiltration using centrifugal filter units with 10-kDa cutoff (Sartorius Stedim SUS Inc., CA) for rodent periurethral injection [24].

### In vitro treatment of human bladder and vaginal cells with pSMC-CM

Vaginal fibroblasts and bladder SMCs were passaged onto 6-well plates at passage 2 at a density of 10,000 cells per cm^2^ in 10% FBS and 90% DMEM. When they reached 90% confluency, medium was changed into basal medium 231 (Invitrogen) supplemented with 0.2% albumin (Sigma-Aldrich, St. Louis, MO) to synchronize for 24 h. The cells were then treated for 48 h with non-concentrated CM collected from P0 and P3 pSMCs at 37 °C in an atmosphere of 95% air and 5% CO_2_, while cells in the control groups were treated with the baseline smooth muscle cell growth medium (SMGS; Invitrogen) supplemented with PDGF preincubated at 37 °C in an atmosphere of 95% air and 5% CO_2_ for 48 h.

### Animal care and generation of SUI rat model

Female immunodeficient Rowett Nude rats (RNU, Charles River Laboratories, Hollister, CA, USA, http://www.criver.com/) weighing 200–250 g were used. Animals were maintained at the Stanford University Research Animal Facility in accordance with Stanford University’s Institutional Animal Care and Use Committee guidelines. Animal experiments were approved by the Institutional Review Board of the Stanford University School of Medicine and the Stanford Administrative Panel of Laboratory Animal Care (APLAC).

The rat model of SUI was established via transabdominal urethrolysis as described by Rodriguez et al. [27]. This SUI rat model showed significantly decreased urethral resistance (by leak point pressure measurements) and urethral smooth muscle damage for at least 8 weeks after surgery [19, 28]. Bilateral ovariectomy was done on the rodents to eliminate the influence of estrus cycle on the ECM metabolism and to simulate an estrogen-deficiency state of menopause [29]. In brief, RNU rats were intraperitoneally anesthetized with ketamine (30 mg/kg) and xylazine (3 mg/kg). The ovaries were exteriorized through a lower abdominal incision. After ovarian vessels were ligated, bilateral ovaries were excised. The bladder and urethra were identified and circumferentially separated from the anterior vaginal wall and pubic bone by sharp dissection, thus causing injury to the urethral sphincter and adjacent vagina.

### Conditioned media peri-urethral injection and tissue collection

The rats were randomly divided into four treatment groups: (1) urethrolysis plus 50× concentrated SMGS (sham-SMGS group, *n* = 7), (2) urethrolysis plus 50× concentrated H9-pSMC-CM (H9 CM group, *n* = 6), (3) urethrolysis plus 50× concentrated Huf5-pSMC-CM (Huf5 CM group, *n* = 7), (4) urethrolysis plus 50× concentrated BIR pSMC-CM (BIR CM group, *n* = 8). Three weeks after urethrolysis, the rats were anesthetized with 3–4% v/v isoflurane and 100 μL of the concentrated pSMC-CM or SMGS were injected in the peri-urethral area (once weekly for 3 weeks) at two sites using a 28.5-gauge insulin syringe [19, 24, 25, 30]. Researchers were blinded to the treatment group allocations. Leak point pressure (LPP) testing to evaluate urethral function was performed 5 weeks after initial injection. The rats were then euthanized, and urethra and adjacent vagina were harvested. The proximal part of the urethra and vagina was embedded in Tissue-Tek O.C.T. compound (Sakura Finetek, Tokyo, Japan, http://www.sakura-finetek.com) for histologic study. The middle part of the urethra and vagina was used for RNA extraction, and the distal part of the urethra and vagina was used for protein extraction.

### Leak point pressure (LPP) measurement

The LPP measurement was used to assess urethral sphincter function. LPP was performed as described by Conway et al. [31]. Investigators performing LPP measurement were blinded to the group assignment of each animal. Briefly, 5 weeks after the injection, the rats were anesthetized with ketamine (30 mg/kg) and xylazine (3 mg/kg). A transvesical catheter with a fire-flared tip was inserted into the bladder dome through a small abdominal incision. The abdominal wall was closed, and the catheter was connected via a three-way stopcock to a 50-ml syringe for filling with methylene blue colored saline and to a pressure transducer (TSD 104A, BIOPAC Systems Inc., CA, USA, http://www.biopac.com) for monitoring bladder pressure. The bladder pressure was amplified and sampled by a biological signal acquisition system (BIOPAC MP 150) and digitalized for computer data collection using Acknowledge acquisition and analysis software (BIOPAC Systems Inc.).

Before LPP testing, the spinal cord was transected at the T8–T10 level to eliminate the voiding reflex mediated by spino-bulbo-spinal pathways. The urethral closure mechanism during urine storage remains intact because urethral contractile reflexes activated by sympathetic and somatic nerves responding to bladder distension are predominantly organized at the lumbosacral spinal cord level. The vertical tilt table/intravesical pressure clamp model was used to measure the LPP. The rat was taped to a board and placed in the vertical position. The 50 ml syringe (reservoir) which was connected to the bladder catheter via the three-way stopcock was then fixed onto a metered vertical pole. Bladder filling was done by manually raising the height of the reservoir by 2–3 cm increments for every 2 min starting from 0 cm, until urinary leakage (methylene blue saline) was observed at the urethral meatus. The bladder pressure (measured by the transducer) at which leakage was observed was recorded as the LPP. LPP is thus a measure of the urethral sphincter pressure against bladder filling. The mean of at least three consecutive LPPs was taken as a data point for each animal.

### RNA extraction and quantitative reverse transcription-polymerase chain reaction

Total RNA of cells and rat tissue was extracted with the RNA-STAT-60 reagent (Tel-Test, Inc., Friendswood, TX, USA). RNA yield was determined using a Nanodrop 2000 spectrophotometer (Thermo Scientific). Total RNA (1 μg) was reverse transcribed into cDNA using the M-MLV reverse transcriptase system (Thermo Scientific). PCR primers were described previously [19, 26], except human TIMP2 (Sense: AAGCGGTCAGTGAGAAGGAA; Anti-sense: GATGTTCAAAGGGCCTGAGA), rat MMP2 (Sense: GTAAAGTATGGGAACGCTGATGGC; Anti-sense: CTTCTCAAAGTTGTACGTGGTGGA), and rat TIMP2 (Sense: ACACGCTTAGCATCACCCAGAA; Anti-sense: CAGTCCATCCAGAGGCACTCAT). Real-time quantitative reverse transcription-polymerase chain reaction (qRT-PCR) was carried out on the Mx3005P Multiplex Quantitative PCR System with MxPro QPCR software (Stratagene, La Jolla, CA, US). Brilliant SYBR Green QPCR Master Mix (Stratagene) was used to perform PCR. GAPDH was used as an endogenous reference against which the different template values were normalized. All PCR reactions were performed in duplicate. The cycle of threshold (Ct) method was used for quantification. Data were analyzed by MxPro QPCR software.

### Western blot assay

Cell culture supernatant was collected and further concentrated (10×) by ultrafiltration using centrifugal filter units with 10-kDa cutoff (Sartorius Stedim SUS Inc., CA). Human bSMCs or vaginal fibroblasts were washed twice with cold PBS and homogenized on ice with a RIPA buffer (50 mM Tris, 150 mM NaCl, 1% NP40, 0.5% deoxycholate, 0.1% SDS, 4 mM EDTA, and 2 mM PMSF, PH 7.4) supplemented with proteinase inhibitor cocktail (Roche Diagnostics GmbH, Basel, Switzerland) and then rotated at 4 °C for 2 days to solubilize the protein more efficiently. Cell debris was removed by centrifugation at 14,000 RPM for 30 min. Protein extraction from rodent tissue was performed [32]. Total protein concentrations of concentrated supernatants and cell lysates were determined using the Bradford method (Bio-Rad, Hercules, CA). The samples were reduced with a sodium dodecyl sulfate (SDS) sample buffer containing 5% of 2-mercaptoethanol and boiled for 7 min. The proteins (20 μg/lane for cell lysates and rat tissue lysates, 100 μg/lane for concentrated supernatants) were subjected to 8–10% (wt/vol) polyacrylamide gels (SDS-PAGE). The gels were blotted onto nitrocellulose membranes (Bio-Rad) in an electrophoretic transfer cell (Bio-Rad). Blots were blocked with 5% nonfat milk at 4 °C overnight and then probed with mouse anti-TIMP-1 antibody (1:1000; Calbiochem, La Jolla, CA), mouse anti-TIMP-2 antibody (1:200; Calbiochem, La Jolla, CA), rabbit anti-collagen III antibody (1:1000; Abcam, Cambridge, MA), goat anti-alpha elastin antibody (1:1000; Abcam, Cambridge, MA), and rabbit anti-human elastin antiserum (1:200; Elastin products company, Owensville, MO) at room temperature for 1 h. After washing three times with phosphate-buffered saline with 0.1% Tween-20, pH 7.4 (PBS-T), the membrane was then incubated with sheep anti-mouse IgG conjugated to HRP (1:5000, GE Healthcare, Pittsburgh, PA) or donkey anti-rabbit IgG conjugated to HRP (1:5000, GE Healthcare) or mouse anti-goat IgG conjugated to HRP (1:5000, GE Healthcare) for 1 h at room temperature, followed by three washes in PBS-T. Blots were developed by chemiluminescence. The blots were re-probed with goat anti-GAPDH polyclonal antibody (1:5000, Abcam) and then dilution of mouse anti-goat IgG conjugated to HRP (1:5000; Invitrogen). Albumin was used as loading control for all the markers tested in the supernatant. The band density was determined by Image Studio Software (LI-COR, Inc., Lincoln, Nebraska USA).

### Elastin staining and qualitative examination of elastin morphology

The proximal urethra and apical vagina were embedded in OCT compound on liquid nitrogen and stored at − 80 °C until cryosectioned. The cryosections were cut in 5-μm thickness and mounted on superfrost slides (Thermo Fisher Scientific Life Sciences). The sections were warmed up for 15 min to room temperature before fixing in 4% wt/vol cold paraformaldehyde (Thermo Fisher Scientific Life Sciences) in PBS (pH 7.4) at room temperature for 15 min. The sections were washed three times in fresh PBS (5 min per wash) to remove OCT and then rinsed in water once. The elastin fibers (black) were stained in Weigert’s Resorcin-Fuchsin solution for 2–4 h according to the manufacturer’s instruction (Electron Microscope Sciences, Hatfield, PA, http://www.electronmicroscopy-sciences.com). The excess solution was removed with 95% vol/vol ethanol. The slides were differentiated with 1% vol/vol acid alcohol and then wash in water. Cell nuclei (dark blue) were stained with Weigert’s iron hematoxylin working solution (Poly Scientific R&D Corporation, Bay Shore, NY, http://www.polyrnd.com) for approximately 30 s. The slides were washed well in running water and then counterstained with van Gieson’s solution for 3–5 min for collagen/elastin fibers (red pink). The excess stain was rinsed from the slides with distilled water.

The slides were visually scored by four people separately for elastin length (1 = short, 2 = moderate, 3 = long), thickness (1 = thin, 2 = moderate, 3 = thick), and density (1 = sparce, 2 = moderate density, 3 = dense), collagen density (1 = sparce, 2 = moderate density, 3 = dense). The scorers were blinded to the group assignments. Scores were totaled for each characteristic and used for comparison to assess whether there were gross, visible differences in the morphologic characteristics [33, 34].

### Immunofluorescence staining

The cryostat sections of rodent tissues were prepared and fixed, as described previously [26]. The slides were treated with 0.25% vol/vol Triton-100 in phosphate-buffered saline (PBS) for 10 min at room temperature. After washing with PBS-Tween (PBS-T; 0.01% vol/vol Tween in PBS) and blocking with 1% wt/vol bovine serum albumin in PBS-T, the slides were incubated with primary antibodies overnight at 4 °C followed by appropriate secondary antibodies in a moisture chamber. Primary antibodies against the following molecules were used: smoothelin (1:50; rabbit polyclonal antibody; Santa Cruz Biotechnology, Santa Cruz, CA, http://www.scbt.com). Secondary antibodies were mouse anti-rabbit IgG-Alexa Fluor 488(1:200; Thermo Fisher Scientific Life Sciences). 4,6-Diamidino-2-phenylindole (DAPI) was used to stain the nuclei. After washing and mounting, the slides were examined with a fluorescence microscope.

### Gelatin zymography

Gelatinolytic activities of matrix metalloproteinases (MMPs) in cell culture supernatants and in cell lysates were assessed by gelatin zymography [35]. In brief, samples were mixed with nonreducing sample buffer before being electrophoresed in 8% polyacrylamide gels containing 0.1% gelatin in the presence of SDS. After electrophoresis, the gels were washed twice with 2.5% Triton X-100 and were subsequently incubated overnight at 37 °C in the substrate buffer (containing 50 mM Tris-HCL, pH 8, 5 mM, CaCl_2_, 0.02% Azide). After staining with Coomassie blue, enzyme activity appeared as clear bands against the blue-stained background. The area of lysis for each band detected was analyzed using Bio-Rad Quality One Software (Bio-Rad). The band density was determined by ImageJ Software (National Institutes of Health, Bethesda, MD, USA).

### Statistical analysis

Statistical analyses were performed using SPSS version 21 (SPSS Inc., Chicago, IL) and JMP version 12 (SAS, Cary, NC). Results are expressed as mean ± SEM. One-way ANOVA followed with Tukey HSD test was used for multiple comparison of the in vitro studies (SPSS). Nonparametric, Kruskal-Wallis one-way ANOVA followed with Wilcoxon method was used for comparison between groups for the in vivo study (JMP). A value of *p* < 0.05 was considered significant.

## Results

### pSMC-CM upregulated extracellular matrix elastin metabolism in human bladder smooth muscle cells (bSMCs) and vaginal fibroblasts

**Fig. 1:**
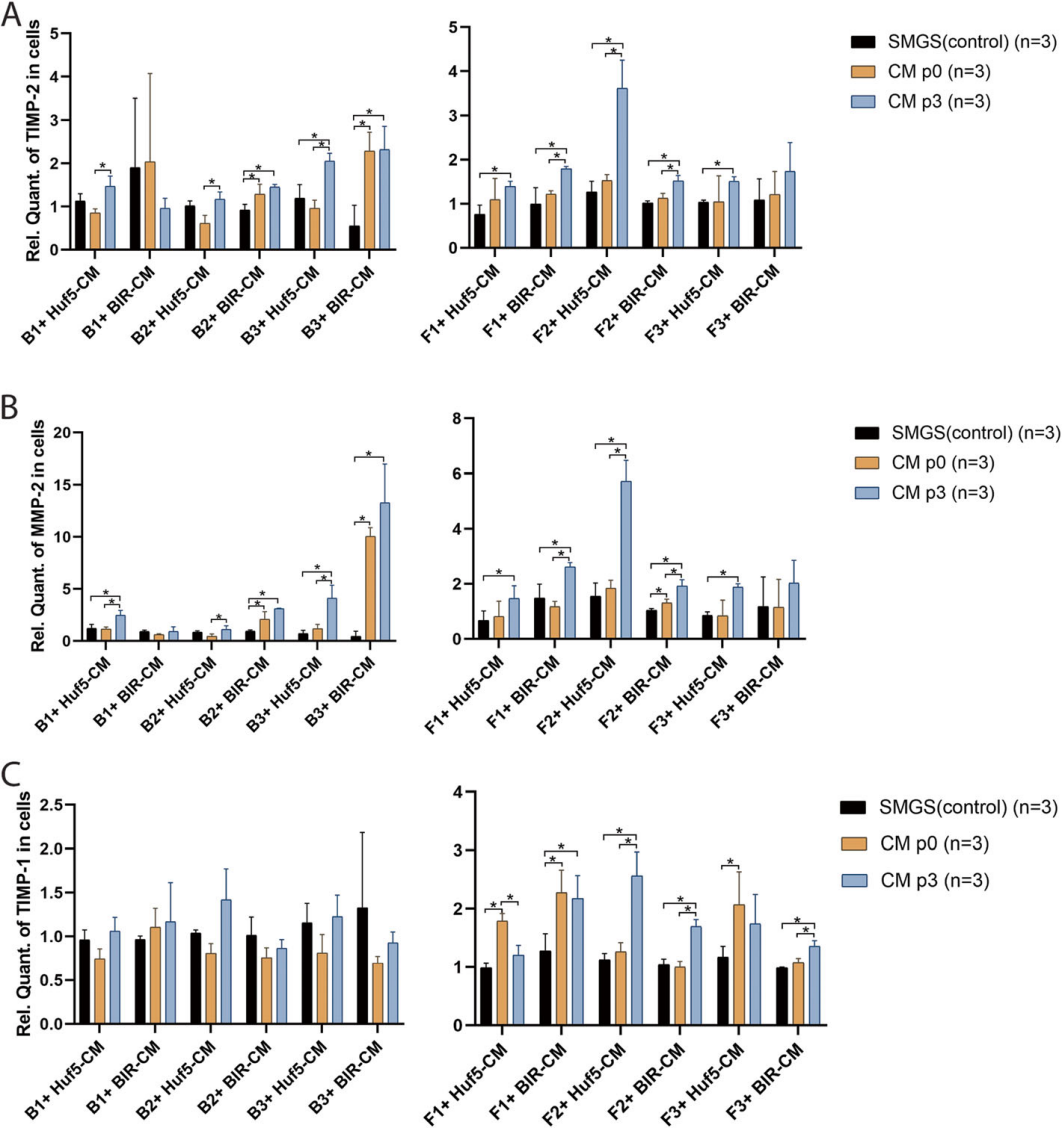
Gene expression of extracellular matrix (ECM) metabolism proteins in pSMC-CM-treated bSMCs and vaginal fibroblasts (**a**–**c**). Gene expression levels of TIMP-2 (**a**), MMP-2 (**b**), and TIMP-1 (**c**) were analyzed by quantitative real-time RT-PCR in bSMCs from three women (B1, B2, B3) and vaginal fibroblasts from three women (F1, F2, F3). Data shown represent the mean ± SD from three independent experiments. Huf5-CM conditioned medium from Huf5 iPSC-derived pSMCs, BIR-CM conditioned medium from BIR iPSC-derived pSMCs, SMGS = SMGS only, no CM treatment (controls). *Significant difference between groups (*p* < 0.05)

To examine how pSMC-CM regulates ECM metabolism in human bladder smooth muscle cells (B1, B2, B3) and vaginal fibroblasts (F1, F2, F3), qPCR was performed on the cells after treatment with pSMC-CM (Fig. 1). Cells from three participants were treated with pSMC-CM derived from two human PSC lines (Huf5 and BIR). Compared to the cells treated with control medium (SMGS supplemented with PDGF, i.e., medium only without the secretomes), 9/12 groups (B1+ Huf5-CM, B2 + BIR-CM, B3 + Huf5-CM, B3 + BIR-CM, F1 + Huf5-CM, F1 + BIR-CM, F2 + Huf5-CM, F2 + BIR-CM, F3 + Huf5-CM) treated with pSMC-CM showed a significant increase in TIMP-2 and MMP-2 mRNA expression (*p* < 0.05) (Fig. 1a, b). TIMP-1 mRNA expression in bSMCs was not significantly different compared to control medium for all Huf5 and BIR pSMC-CM; while in 4/6 fibroblasts groups (F1 + BIR-CM, F2 + Huf5-CM, F2 + BIR-CM, F3 + BIR-CM), mRNA expression of TIMP-1 was significantly increased by P3-pSMC-CM (*p* < 0.05) (Fig. 1c).

**Fig. 2:**
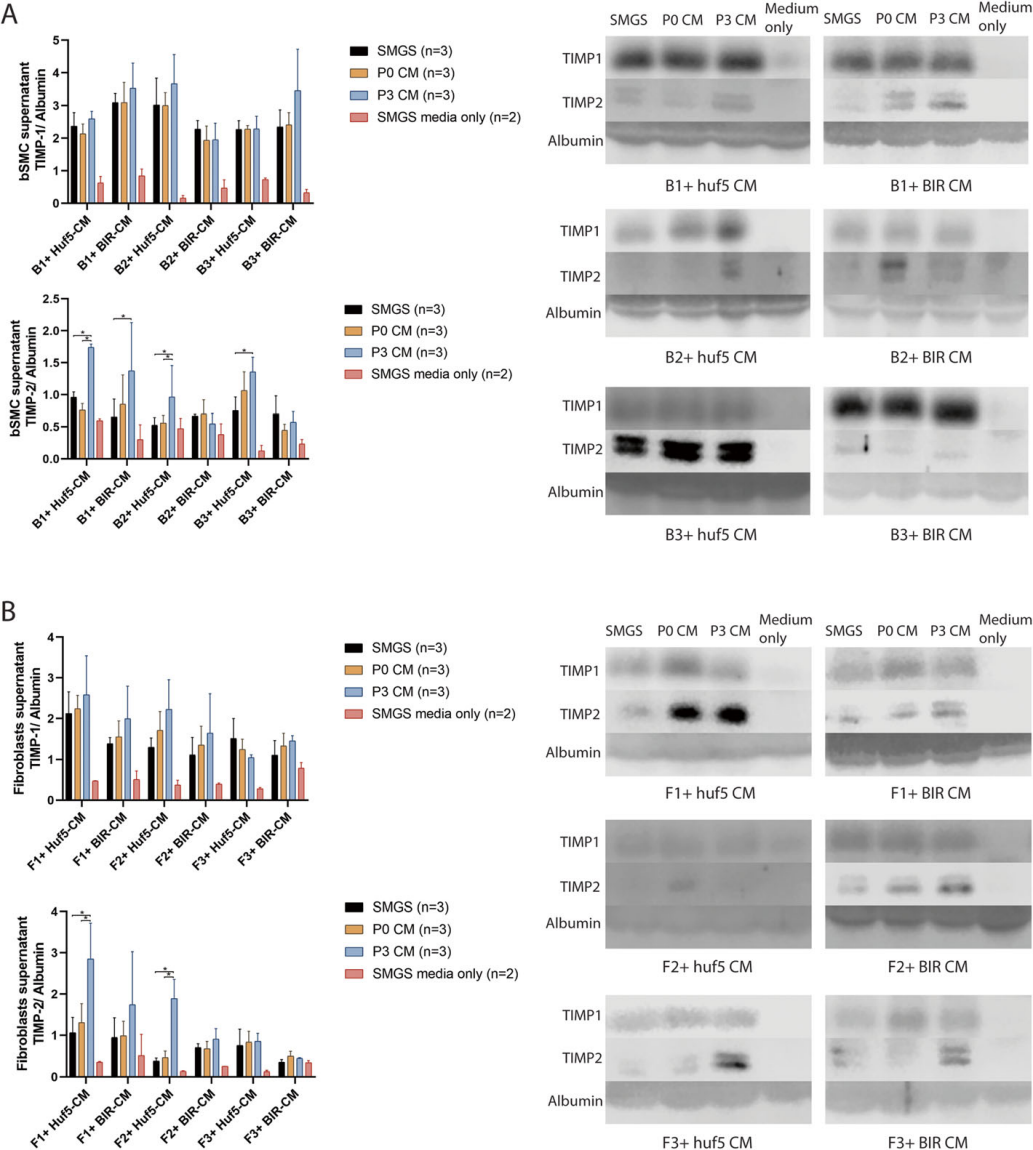
TIMP-1 and TIMP-2 protein expression in the supernatant of the bSMCs (**a**) and vaginal fibroblasts (**b**) treated with pSMC-CM. Each bar plot represents 6 separate experiments run on 6 gels to illustrate the statistical trends for each experiment. Each experiment consists of the SMGS-media-only, SMGS, CM at P0, and CM at P3 groups (black, orange, blue, and pink bars). The expression levels for each bar can be compared within each experiment, but not between experiments. Data shown represent the mean ± SD from three independent experiments. Huf5-CM conditioned medium from Huf5 iPSC-derived pSMCs, BIR-CM conditioned medium from BIR iPSC-derived pSMCs, *SMGS* supernatant from bSMC or vaginal fibroblasts treated with SMGS only, SMGS media only = concentrated SMGS media only. *Significant difference between groups (*p* < 0.05)

Western blot was performed to examine TIMP-1 and TIMP-2 secreted by cells in the supernatant (Fig. 2). In 4/6 bSMCs and in 2/6 fibroblast groups, P3 pSMC-CM-treated cells secreted more TIMP-2 than the control medium treated cells (*p* < 0.05). Neither bSMCs nor fibroblasts appear to secrete TIMP-1 when cultured with passage 0 or passage 3 pSMC-CM (Fig. 2a, b).

**Fig. 3:**
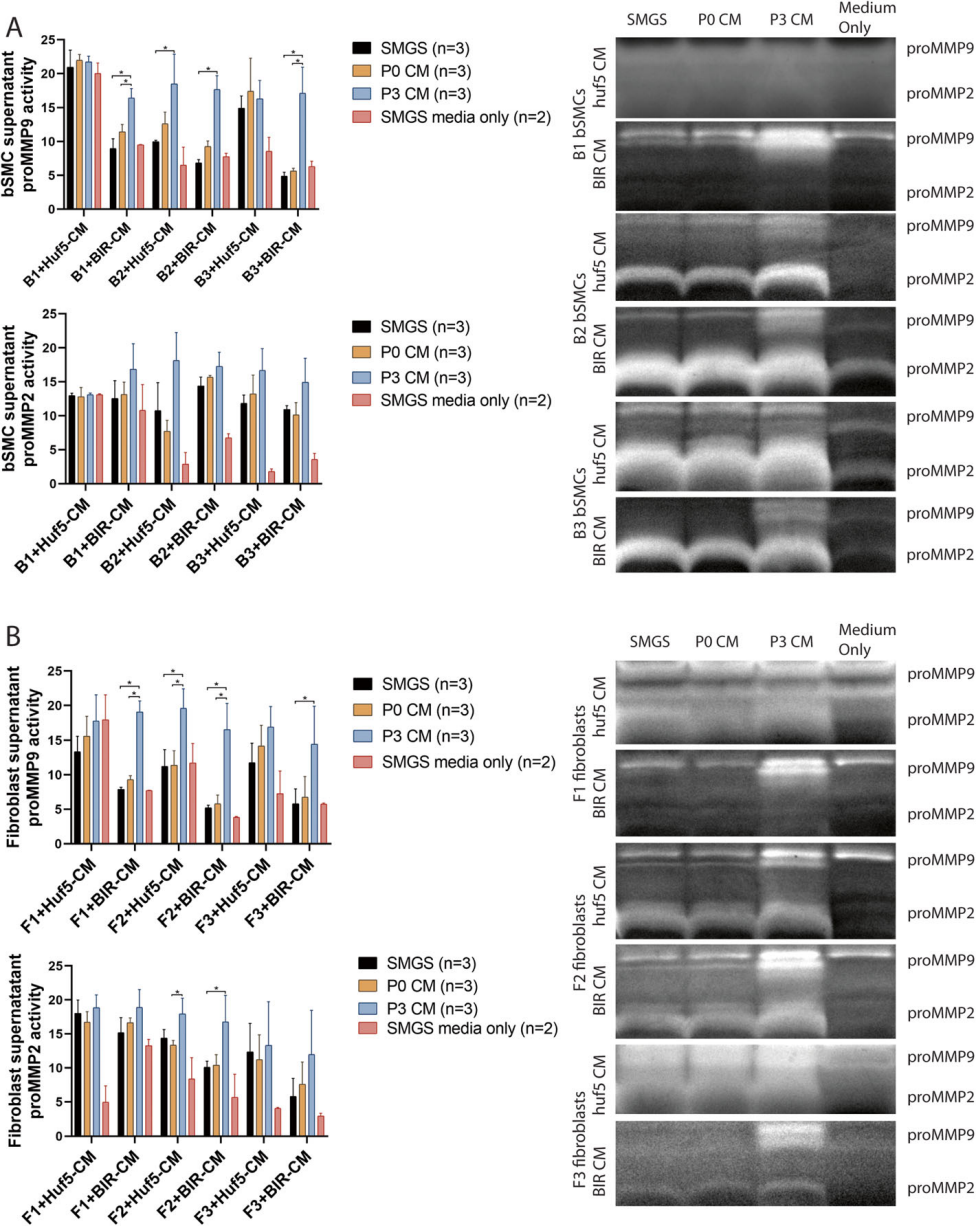
pro-MMP2 and pro-MMP9 activity of pSMC-CM-treated bSMCs (**a**) and vaginal fibroblasts (**b**). Zymographic evaluation of MMP activities in concentrated supernatant from bSMCs and vaginal fibroblasts treated with different pSMC-CM. Each bar plot represents 6 separate experiments run on 6 gels to illustrate the statistical trends for each experiment. Each experiment consists of the SMGS-media-only, SMGS, CM at P0, and CM at P3 groups (black, orange, blue, and pink bars). The expression levels for each bar can be compared within each experiment, but not between experiments. Data shown represent the mean ± SD from three independent experiments. Huf5 CM conditioned medium from Huf5 iPSC-derived pSMCs, BIR CM conditioned medium from BIR iPSC-derived pSMCs, SMGS supernatant from bSMC or fibroblasts treated with SMGS only, SMGS media only = concentrated SMGS media only, not the supernatant from cell culture. *Significant difference between groups (*p* < 0.05)

The activities of pro-MMPs (MMPs’ zymogen form) in concentrated cell culture supernatants were examined by nonreducing gelatin zymography (Fig. 3). In 8/12 groups, passage 3 pSMC-CM significantly upregulated pro-MMP-9 activity in the supernatant of the treated cells (*p* < 0.05). pro-MMP-2 activity was similar to controls in all groups (Fig. 3).

### pSMC-CM promoted gene expression of collagen and elastin in bSMCs and vaginal fibroblasts

**Fig. 4:**
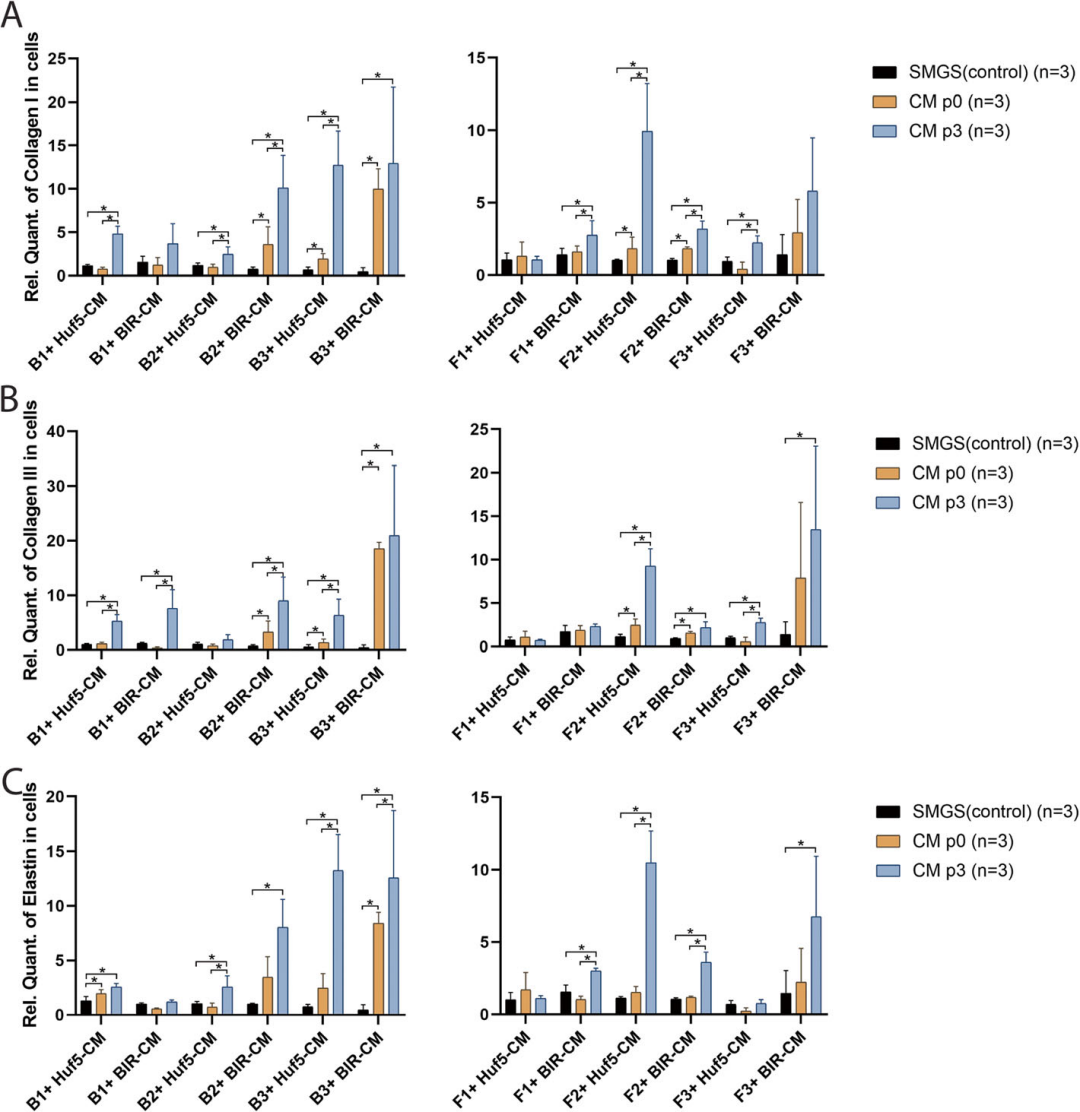
Gene expression of collagen I, collagen III, and elastin in conditioned medium-treated bSMCs and vaginal fibroblasts (**a**–**c**). Data shown represent the mean ± SD from three independent experiments. Huf5 CM conditioned medium from Huf5 iPSC-derived pSMCs, BIR CM conditioned medium from BIR iPSC-derived pSMCs, SMGS bSMC or fibroblasts treated with SMGS only (controls). *Significant difference between groups (*p* < 0.05)

Similar to the ECM metabolism protease data reported above, passage 3 pSMC-CM significantly increased gene expression of collagen I, collagen III, and elastin in 9/12 groups (Fig. 4). However, upregulation of collagen I, collagen III, and elastin protein expression was only observed for collagen III in B3 + BIR-CM, F1 + Huf5-CM, and F1 + BIR-CM (data not shown).

### *pSMC-CM* improved urethral leak point pressure *in a stress urinary incontinence rat model*

**Fig. 5:**
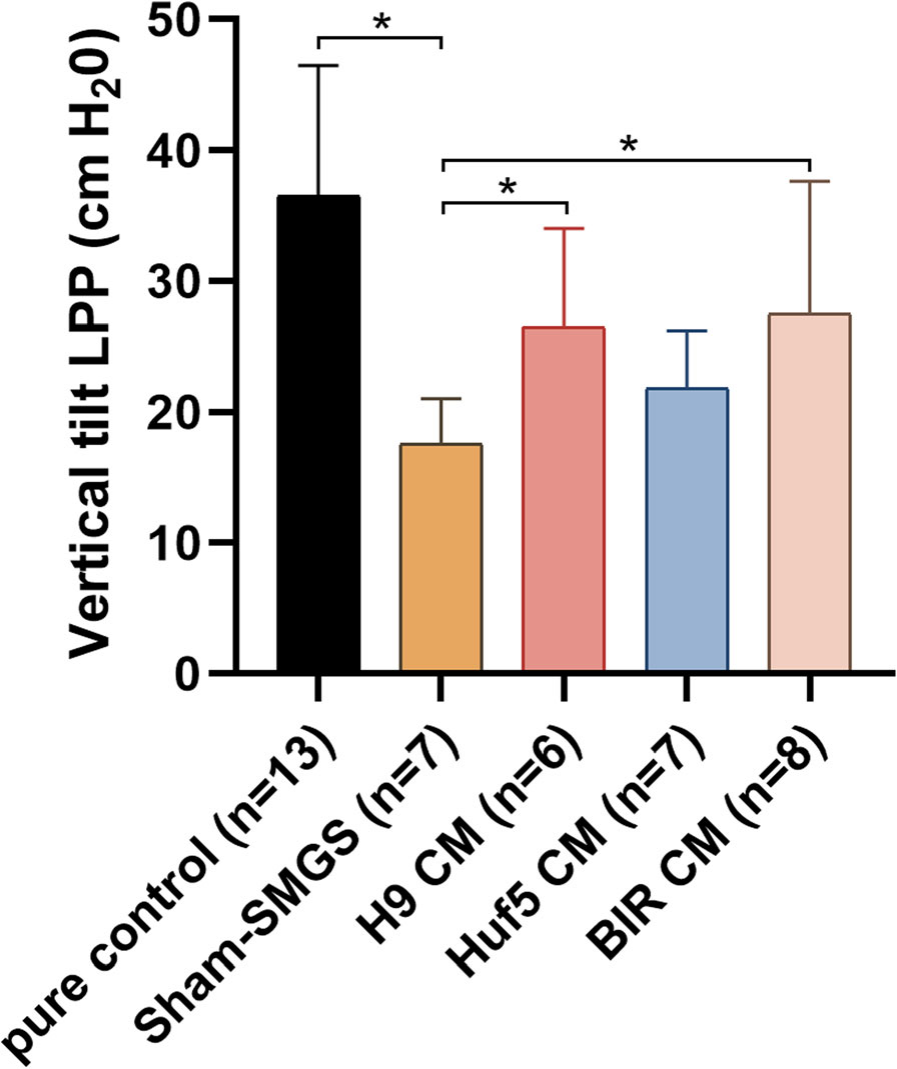
Comparison of leak point pressure (LPP) values of SUI rats in different groups. Data shown represent the mean ± SD. Pure control = rats with no surgery and no treatment; Sham SMGS = SUI rats treated with concentrated SMGS only; H9 CM = SUI rats treated with conditioned medium from H9 ESC-derived pSMC; Huf5 CM = SUI rats treated with conditioned medium from Huf5 iPSC-derived pSMCs; BIR CM = SUI rats treated with conditioned medium from Huf5 iPSC-derived pSMCs. *Significant difference between groups (*p* < 0.05)

Stress urinary incontinence (SUI) was induced in rats by surgical urethrolysis. Leak point pressure of the urethra (LPP) was used to evaluate in vivo urethral function after peri-urethral injection of the SUI rats with pSMC-CM derived from three human pluripotent stem lines: two iPSC lines (Huf5, BIR) and one embryonic stem cell line (H9). Compared with pure control rats (no surgery and no pSMC-CM treatment), the mean LPP was significantly lower in the SUI rats treated SMGS (sham controls) 8 weeks after urethrolysis (17.59 ± 3.18 cm H_2_O vs. 36.51 ± 9.58 cm H_2_O, *p* < 0.05), indicative of persistent decreased urethral function in the sham controls. Two out of three SUI rat groups treated with pSMC-CM (H9-pSMC-CM and BIR-pSMC-CM) showed significantly higher LPP compared to sham-SMGS rats (26.5 ± 6.85 cm H_2_O vs. 17.59 ± 3.18 cm H_2_O, 27.5 ± 9.50 cm H_2_O vs. 17.59 ± 3.18 cm H_2_O, Fig. 5).

### pSMC-CM induced ECM remodeling in the rat urethra

**Fig. 6:**
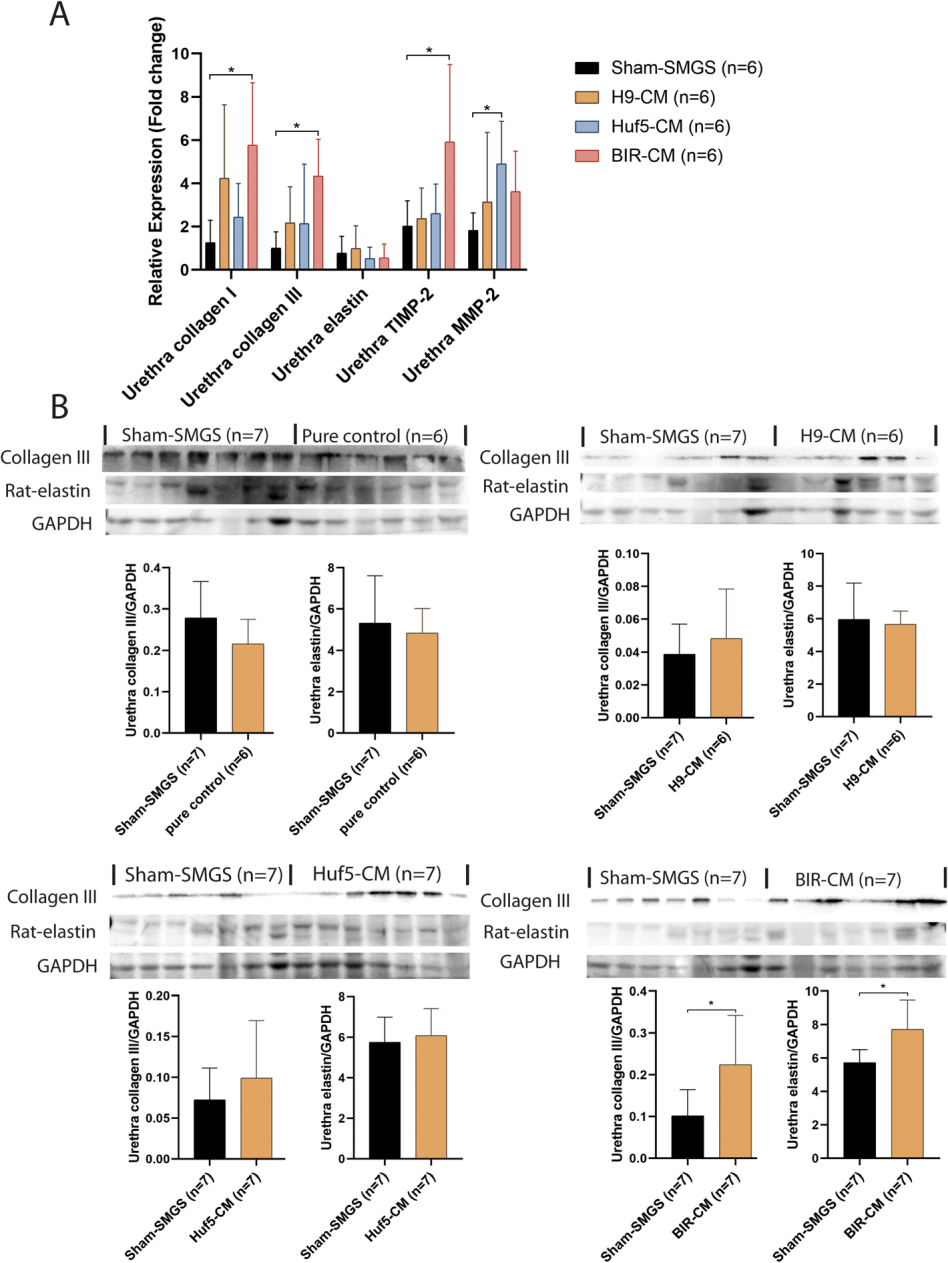
Gene expression of rat-collagen I, rat-collagen III, rat-elastin, TIMP-2, and MMP-2 (**a**) and protein expression of rat-collagen III and rat-elastin in conditioned medium treated rat urethras for each group (**b**). Data shown represent the mean ± SD. Pure control = rats with no surgery and no treatment; Sham SMGS = SUI rats treated with concentrated SMGS only; H9 CM = SUI rats treated with conditioned medium from H9 ESC-derived pSMC; Huf5 CM = SUI rats treated with conditioned medium from Huf5 iPSC-derived pSMCs; BIR CM = SUI rats treated with conditioned medium from Huf5 iPSC-derived pSMCs. *Significant difference between groups (p < 0.05)

Our previous study documented that alteration of ECM components in the lower urinary tract after urethrolysis was associated with the development of urine incontinence in SUI rats [19]. To evaluate the effect of pSMC-CM on in vivo ECM remodeling in the lower urinary tract, we examined expression of collagen I, collagen III, elastin, TIMP-2, and MMP-2 in rat urethra and vagina. pSMC-CM treated rats demonstrated increased collagen I, collagen III, and TIMP-2 mRNA expression in the BIR pSMC-CM compared to sham-SMGS group (Fig. 6a). This was reflected in an increased expression of collagen III and elastin protein in the urethra (Fig. 6b), suggesting that injection of pSMC-CM induced ECM remodeling.

Elastin morphology of pSMC-CM-treated rat urethra was qualitatively described by visual scoring done by 4 observers blinded to the study groups. Compared with sham-SMGS, elastic fibers in urethras of the rats treated with H9-pSMC-CM and BIR-pSMC-CM were observed to be thicker, longer, and denser. Rats from H9-pSMC-CM and BIR-pSMC-CM group also had denser collagen content in the urethra compared to the sham-SMGS group (see Supplemental File, Figure 2).

### pSMC-CM induced ECM remodeling and the smooth muscle cell layer changes in SUI rat vagina

**Fig. 7:**
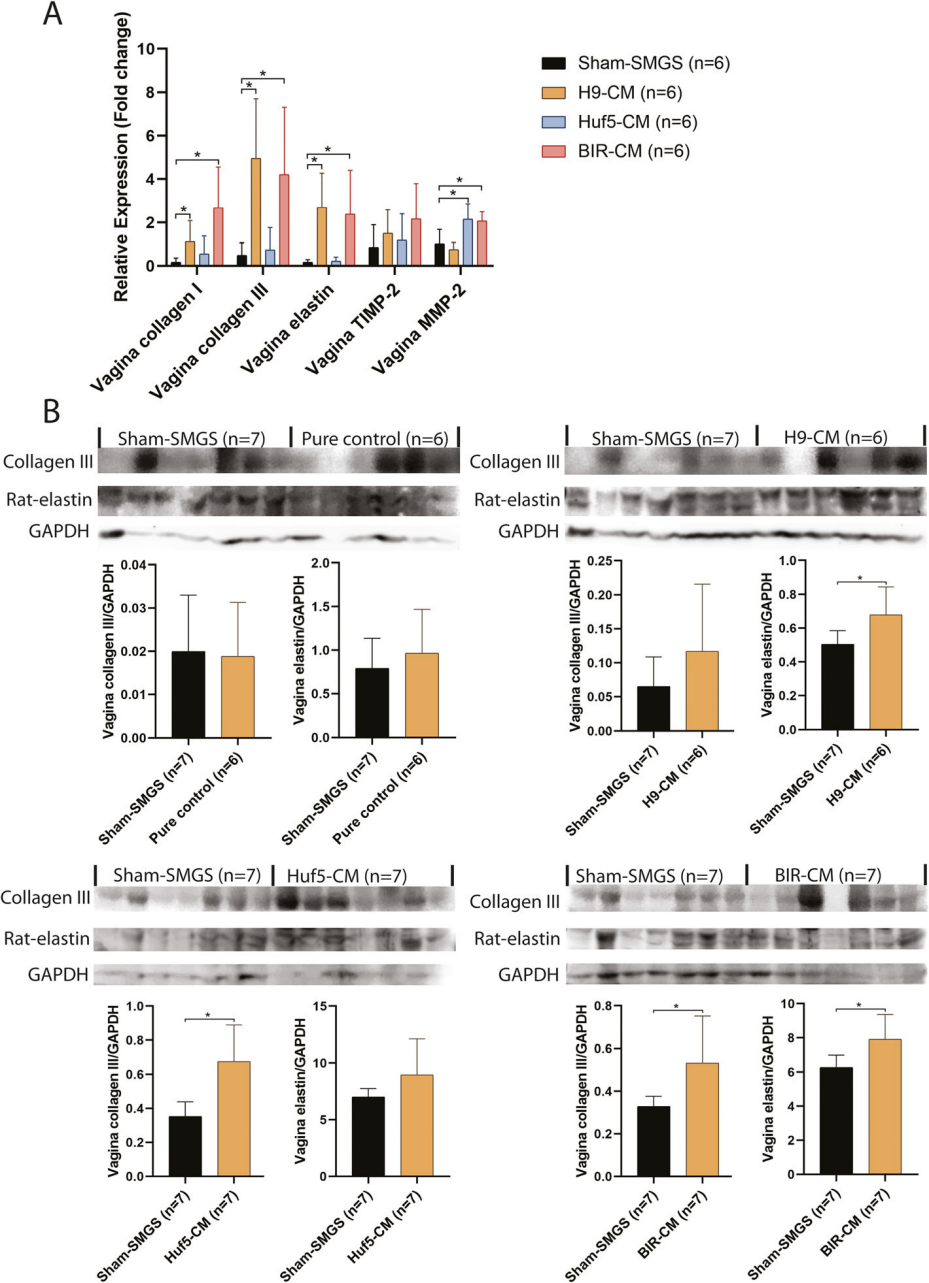
Gene expression of rat-collagen I, rat-collagen III, rat-elastin, TIMP-2, and MMP-2 (**a**) and protein expression of rat-collagen III and rat-elastin in conditioned medium treated rat vaginas (**b**). Data shown represent the mean ± SD. Pure control = rats without surgery and no treatment; Sham SMGS = SUI rats treated with concentrated SMGS; H9 CM = SUI rats treated with conditioned medium from H9 ESC-derived pSMC; Huf5 CM = SUI rats treated with conditioned medium from Huf5 iPSC-derived pSMCs; BIR CM = SUI rats treated with conditioned medium from Huf5 iPSC-derived pSMCs. *Significant difference between groups (*p* < 0.05)

Vaginal prolapse is frequently associated with SUI [36], it is thought that the connective tissue and vaginal wall under the urethra provide support and contribute to the urinary continence mechanism. Therefore, we examined ECM remodeling in the rat vagina which is in close proximity to the injection site. H9-pSMC-CM and BIR-pSMC-CM-treated SUI rat vaginas had significantly higher mRNA expression of collagen I, collagen III, and elastin compared to the SUI rat vaginas from the sham-SMGS group. mRNA expression of MMP-2 in the vagina was found to be significantly increased in Huf5-pSMC-CM and BIR-pSMC-CM groups compared with the sham-SMGS group (Fig. 7a).

Collagen III and elastin protein expression in the vagina was also examined. Collagen III expression was found to be significantly higher in Huf5-pSMC-CM and BIR-pSMC-CM treated rats, and elastin expression was found significantly higher in H9-pSMC-CM and BIR-pSMC-CM-treated rats compared with sham-SMGS treated rats (Fig. 7b).

**Fig. 8:**
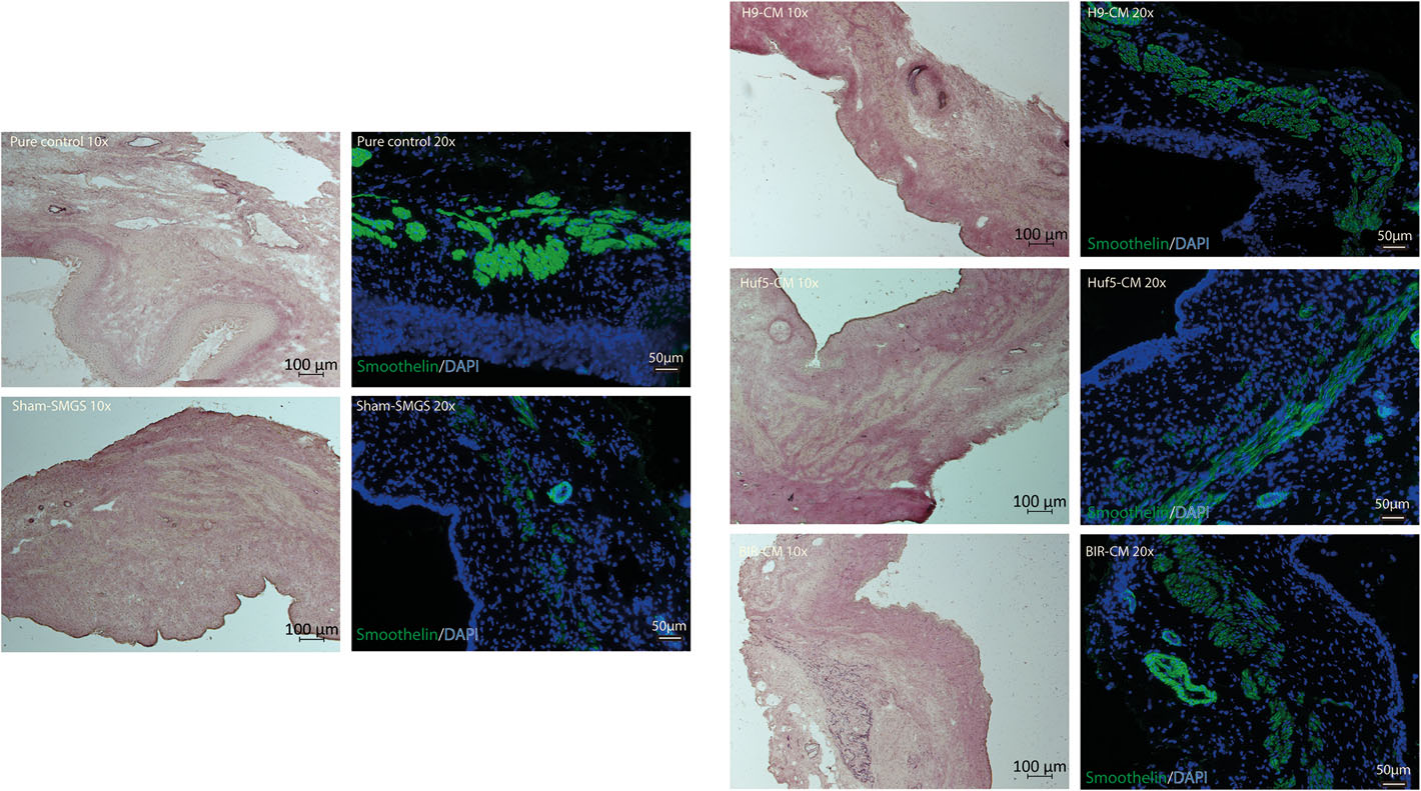
Effect of pSMC-CM on the muscle layer of the rat apical vagina wall. Representative images of cross-section of proximal vagina (apical vagina). Pink stains are tissue stains for collagen/elastin to show tissue structure. Green/blue stains are immunofluorescence stains of the same section with smoothelin staining for smooth muscle cells (green) and DAPI stain for cell nucleus (blue). Pure control = rats with no surgery and no treatment; Sham SMGS = SUI rats treated with concentrated SMGS only; H9 CM = SUI rats treated with conditioned medium from H9 ESC-derived pSMC; Huf5 CM = SUI rats treated with conditioned medium from Huf5 iPSC-derived pSMCs; BIR CM = SUI rats treated with conditioned medium from Huf5 iPSC-derived pSMCs

Three samples of vaginal wall tissue were randomly selected from each group and stained by immunofluorescence for smoothelin (a smooth muscle cell protein) to assess whether the injected pSMC-CM induced a myogenic response. Rats in the sham-SMGS group exhibited disrupted and thinner smooth muscle layer compared with the pure control group (no surgery or treatment), consistent with smooth muscle cell destruction due to the surgical urethrolysis. More robust smoothelin expression was observed in the pSMC-CM treated groups (Fig. 8).

## Discussion

SUI is thought to arise from the decreased support of the pelvic floor and vaginal connective tissue around the bladder neck and urethra [36], and loss of urethral sphincter muscle [37]. Current surgical treatment is efficacious, but the adverse events associated with surgery and permanent synthetic meshes implants, postoperative voiding dysfunction [11, 12], and long-term recurrence rates have led to a quest for the development of new non-surgical approaches.

Several studies have suggested that a key mechanism by which transplanted adult MSCs contribute to tissue repair is through their paracrine function [38–41]. Our studies using smooth muscle cell progenitors (pSMC) differentiated from human iPSCs showed that pSMC transplantation into the urethra of the SUI rat model also yielded histologic evidence of extracellular remodeling in the native rodent tissue [19, 30].

While these data suggest an in vivo paracrine effect of pSMCs in the SUI rodent model, the long term, in vivo effects of secretomes from PSC-derived progenitor cells have never been tested. In the present study, we sought to further investigate the paracrine effect of human smooth muscle cell progenitors (pSMCs) on the extracellular matrix metabolism and test whether administration of pSMC-conditioned media alone, without the cells, can restore urethral function.

Our previous studies showed that peri-urethral tissues from SUI women exhibit differential expression of MMP/TIMP compared to tissues from asymptomatic women [42–46]. Other investigators have also documented that differential MMP or TIMP expression is associated with pelvic floor disorders [47, 48]. In tissues, collagen provides strength while elastin provides elasticity and the ability to recoil. Both are important in conferring to tissue its mechanical properties. Hence, we assessed the above proteins to evaluate ECM metabolism.

Conditioned media from passaging of pSMCs, which contains the cell culture medium and the cell secretomes, were used to evaluate the in vitro effect of pSMCs on cells from the bladder and the vagina. Our data show that in bSMCs, pSMC-CM upregulated gene expression of extracellular matrix proteins MMP-2 and TIMP-2, while it did not affect TIMP-1 gene expression. In vaginal fibroblasts, pSMC-CM upregulated gene expression of MMP-2, TIMP-1, and TIMP-2. Enzyme activity assay revealed that MMP-9 activity was increased with no significant change in MMP-2 activity in cells treated with pSMC-CM compared to controls. Taken together, these in vitro alterations induced by pSMC-CM treatment suggest modulation of elastin in the ECM, as MMP-2, MMP-9, and TIMP-2 are proteases and anti-proteases involved in elastin metabolism. Moreover, gene expression of the main structural components of the ECM (collagen I, collagen III, and elastin) which contribute to the biomechanical properties of lower urinary tract soft tissue were all found to be upregulated in the cells after treatment with pSMC-CM. We believe that the lack of increased collagen and elastin protein expression is due to the short treatment period (48 h) which may not enough time for synthesis of more complex proteins. It appears that, generally, passage 3 pSMC-CM has a stronger effect than passage 0 CM. We think this is due to our differentiation protocol, P0 pSMCs are at their first passage post FACS sorting. At this stage, they are still mostly epithelial progenitor cells. After they are passed to P3, most of the cells are more specifically differentiated into the smooth muscle cell progenitor (pSMC) phenotype. This is also the stage that showed in vivo effect of ECM changes in our previous experiment using pSMC transplantation into the SUI rat [19].

We used an established SUI rat model to test whether pSMC-CM would result in improvement of the leak point pressure of the urethra (LPP). The in vivo data showed significant increase in LPP with pSMC-CM treatment compared to sham controls. The SUI rats injected with pSMC-CM also showed ECM remodeling with higher local elastin and collagen expression. Vaginal prolapse is frequently associated with SUI, it is thought that the connective tissue and the vaginal wall under the urethra provide support and contribute to the urinary continence mechanism [36]. Interestingly, the smooth muscle layers in the treated vagina adjacent to the urethra appeared thickened compared to sham treatment. Proliferation of the native smooth muscle cells was not evaluated in this study. Whether CM stimulates smooth muscle cell regeneration should be further evaluated.

Taken together, data from this present study suggest that pSMC-CM may contribute to the effect of the iPSC-derived smooth muscle cell progenitors on urethral LPP and may be sufficient for LLP improvement when used alone without cell transplantation. Further studies will be needed to confirm the therapeutic effect of human PSC-derived conditioned media for stress urinary incontinence.

Limitations of this study include limited sample size and limited ability to detect proteins present in low concentrations. The small samples also precluded our ability to examine a wider range of proteins. We acknowledge that there are likely other growth factors such as transfer growth factors-β (TGF-β), vascular endothelial-derived growth factor (VEGF), platelet-derived growth factor (PDGF), and epidermal growth factor (EGF) that may be involved but were not detected by our assays. We also note that more comprehensive studies to document other proteins and pathways in the EMC, as well as assessment of smooth muscle cell component, are needed to fully elucidate the effect of CM on urethral function.

To our knowledge, this is the first study to document that CM from smooth muscle cell progenitors derived from human PSC lines can exert a functional and histological effect on the urethra and vagina. These data support future development efforts for iPSC as stem cell source for progenitor cell-CM therapies for treatment of pelvic floor disorder. This novel treatment would bypass many of the regulatory safety issues associated with cell therapy. Additionally, a major advantage of this approach over using an adult MSC source is the ability to differentiate large homogenous progenitor cell populations for reproducible CM production. The CM can be produced and stored in quantities sufficient for multi-dose applications to improve efficacy over time or for prevention of tissue deterioration in pelvic floor disorders.

## Conclusion

Conditioned media from smooth muscle cell progenitors derived from human pluripotent stem cells improve urethral leak point pressure in vivo. This is accompanied by ECM remodeling through upregulation of MMP-2, MMP-9, TIMP-2, and collagen I, collagen III, and elastin expression in cells (in vitro) and tissue (in vivo) from the urethra and adjacent vagina. These findings support future studies to investigate CM as a novel therapy for PSC-based treatments for pelvic floor disorders where tissues are affected by elastin and smooth muscle loss.

## Supplementary Information

**Additional file 1: Supplemental File Figure 1.** Gene expression of collagen I, collagen III and elastin in conditioned medium-treated bSMCs and vaginal fibroblasts **(A-C).** Each point represents individual value from the experiments. Huf5 CM = conditioned medium from Huf5 iPSC-derived pSMCs; BIR CM = conditioned medium from BIR iPSC-derived pSMCs; SMGS = bSMC or fibroblasts treated with SMGS only (controls). * = significant difference between groups (*p* < 0.05).

**Additional file 2: Supplemental File Figure 2.** Elastin fibers in the proximal urethra of the rat. Representative images of cross-section of proximal urethra with Weigert’s Resorcin-Fuchsin’s elastin and van Gieson’s collagen staining. Elastic fiber shown as dark black, collagen as red pink, and other tissue elements as yellow. Red arrows indicate representative elastin fibers in each image. **(A)** Total visual score of each morphologic characteristic of collagen and elastin fibers (**B**). Pure control = rats with no surgery and no treatment; Sham SMGS = SUI rats treated with concentrated SMGS only; H9 CM = SUI rats treated with conditioned medium from H9 ESC-derived pSMC; Huf5 CM = SUI rats treated with conditioned medium from Huf5 iPSC-derived pSMCs; BIR CM = SUI rats treated with conditioned medium from Huf5 iPSC-derived pSMCs.

**Additional file 3.** The example of our data analysis using SPSS software

**Additional file 4.** The example of our data analyses using JMP software

## Data Availability

The datasets used and/or analyzed during the current study are available from the corresponding author on reasonable request.

## Abbreviations

pSMC: Smooth muscle cell progenitors
iPSC: Induced pluripotent stem cells
hESC: Human embryonic stem cell
MSC: Mesenchymal stem cell
ECM: Extracellular matrix
SUI: Stress urinary incontinence
pSMC-CM: Smooth muscle cell progenitors conditioned media
bSMC: Bladder smooth muscle cell
MMP: Matrix metalloproteinase
TIMP: Tissue inhibitor of metalloproteinase
bFGF: Basic fibroblast growth factor
VEGF: Vascular endothelial growth factor
PBST: Phosphate-buffered saline with 0.1% Tween-20
DMEM: Dulbecco’s modified Eagle’s medium
CDM: Chemical defined medium
MACS: Magnetic-activated cell sorting
FACS: Fluorescence-activated cell sorting
VPCs: Vascular progenitor cells
SMGS: Smooth muscle cell growth medium
LPP: Leak point pressure
qRT-PCR: Quantitative reverse transcription-polymerase chain reaction
PDGF: Platelet-derived growth factor
