# Supplementary figures and images for: Secretomes of human pluripotent stem cell-derived smooth muscle cell progenitors upregulate extracellular matrix metabolism in the lower urinary tract and vagina
Source: Stem Cell Res Ther. 2021 Apr 6;12:228. doi: 10.1186/s13287-021-02292-y (PMC8025391; doi:10.1186/s13287-021-02292-y)

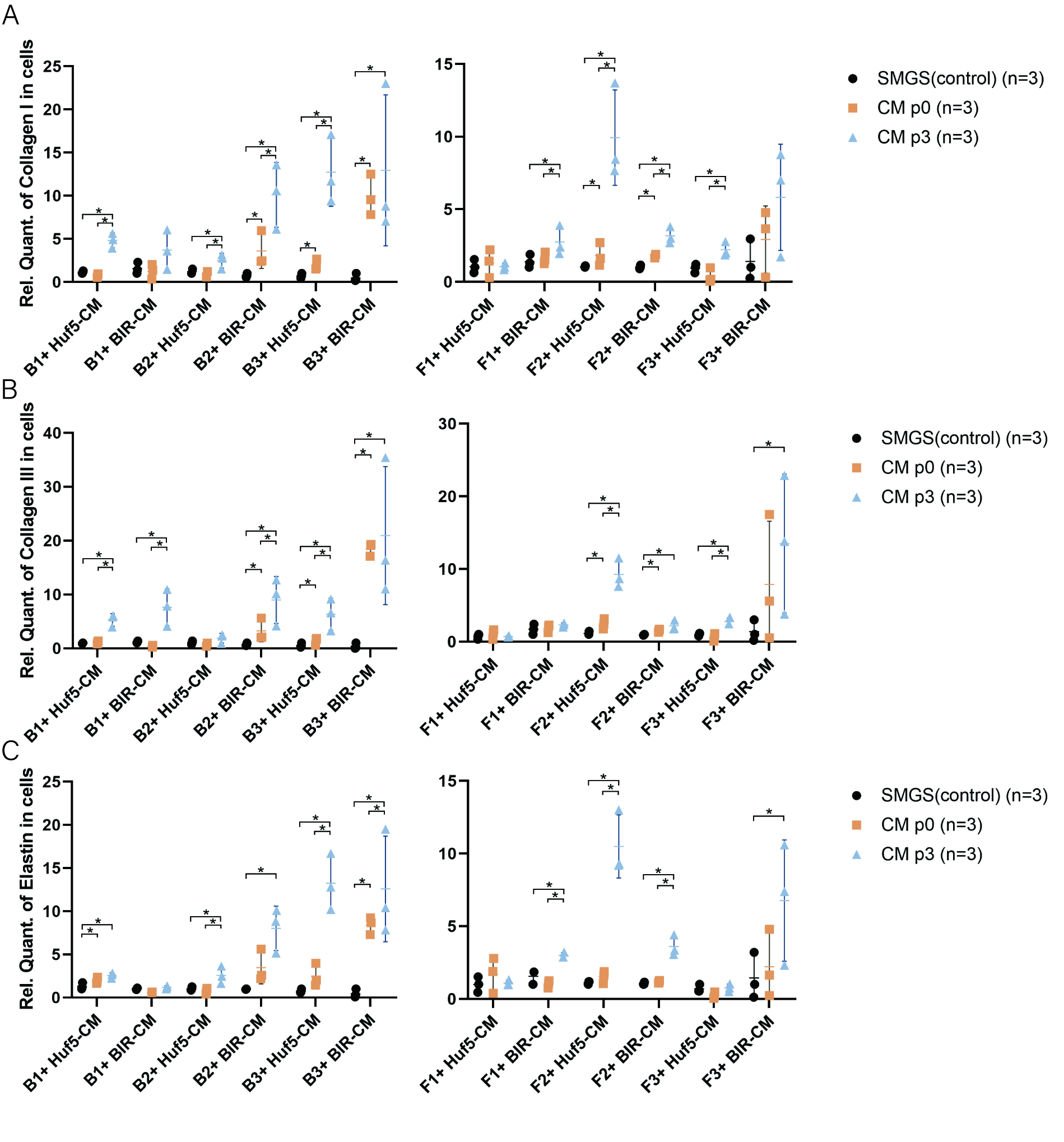

Supplement: Supplementary file 1 — Additional file 1: Supplemental File Figure 1. Gene expression of collagen I, collagen III and elastin in conditioned medium-treated bSMCs and vaginal fibroblasts (A-C). Each point represents individual value from the experiments. Huf5 CM = conditioned medium from Huf5 iPSC-derived pSMCs; BIR CM = conditioned medium from BIR iPSC-derived pSMCs; SMGS = bSMC or fibroblasts treated with SMGS only (controls). * = significant difference between groups (p < 0.05). [file 13287_2021_2292_MOESM1_ESM.tif]

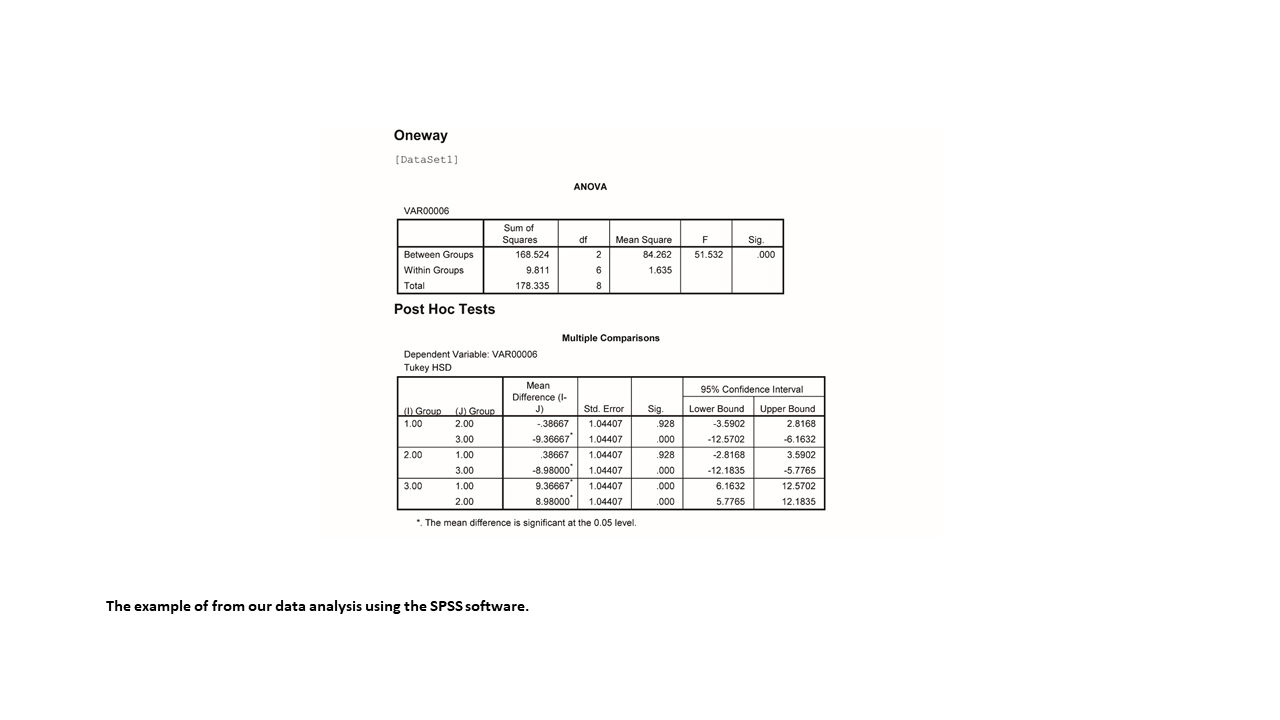

Supplement: Supplementary file 3 — Additional file 3. The example of our data analysis using SPSS software [file 13287_2021_2292_MOESM3_ESM.tif]

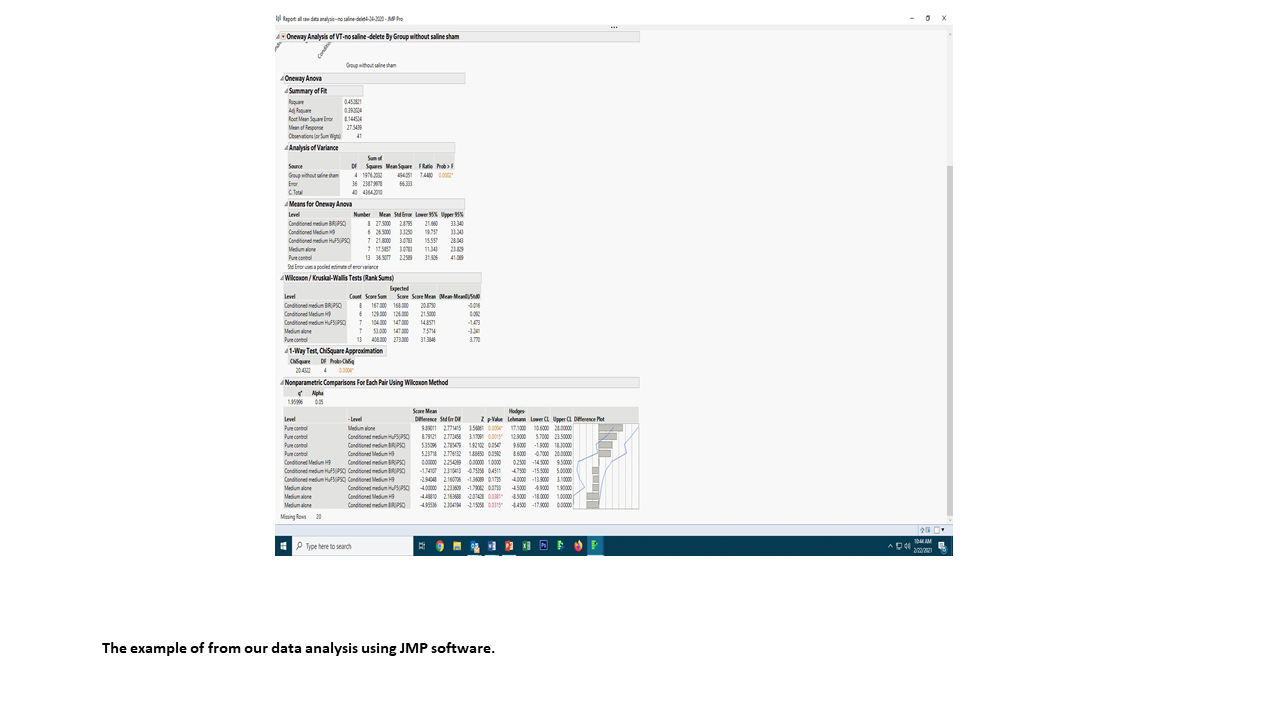

Supplement: Supplementary file 4 — Additional file 4. The example of our data analyses using JMP software [file 13287_2021_2292_MOESM4_ESM.tif]
